# City and Provincial Appointments

**Published:** 1909-02-06

**Authors:** 


### Resident Medical Officers' Department.

### CITY AND PROVINCIAL APPOINTMENTS.

To the newly qualified practitioner one of the most important questions is "What is the best thing to do?" Having graduated, after five or six years of study, he learns now that he has acquired a good theoretical knowledge of his work, but that the dexterity of practice is wanting.

Having decided to take a resident post, the young graduate has a choice of two courses. He may either apply for a resident post in a large city hospital with a medical school attached to it, or for an appointment in one of the small provincial hospitals. It may be of use to consider the advantages and disadvantages of these respective courses.

To begin with, a post in city hospitals is usually an honorary one. In some the junior house surgeon or house physician does not even reside in hospital, but has to pay for his board and lodging outside. He spends eight hours or more at the hospital each day, and pursues an endless round of routine observations, and the endless minutiæ that go to the preparing of material for daily cliniques and lectures. When he attains the dignity of senior house surgeon or physician he resides in the hospital and receives board and perhaps his washing. His work becomes heavier; he continues the daily routine observations, has to write up the cases incessantly, has to control the vagaries of students, and has the anxiety of collecting all the material for the ward cliniques. No doubt he will thus attain proficiency in powers of observation, and will be able to perfect himself in routine medical and surgical work; but it means a further drain on the financial resources of his guardians.

The appointments in such hospitals are usually for six months, and, that being so, at the end of a year he finds that he has gained experience in one branch of his profession. If he has been a house surgeon and wishes to work in the medical wards he has to spend another year in the hospital. Again, if he wishes to obtain experience in special departments, such as eye, ear, nose, and throat, etc., a further period has to be spent in the hospital. In addition to those advantages already mentioned, there are further ones in that he will be associated with a surgeon or a physician of outstanding ability, and if he desires to undertake research work there will be more material in a large city hospital. The duties are often arduous and exacting, and the resident may have considerable difficulty in finding time for exercise and recreation.

Contrast with this the post of resident in a small provincial hospital. Perhaps he may be the only resident, or perhaps there may be two or more: in any case as senior he would have the control of the work of the hospital as a whole. The experience that can be gained in a provincial hospital is most extensive. Cases of all kinds (medical, surgical, and gynæcological) are received and treated, and in many hospitals there is a children's ward attached, so that experience in this most important branch can be got. Again, dispensing may fall to his lot; and the capital training in dispensing obtainable in some hospitals may prove of the utmost value afterwards. The administration of anæsthetics will probably be another duty, and there can be no doubt that adequate experience in this branch of work is invaluable before embarking on general practice. At the present time many of the provincial hospitals have an $x$-ray department, and the resident in some cases will be expected to acquire a knowledge of the working of the apparatus, so that he can, in the absence of the radiographer, make use of it for diagnostic or therapeutic purposes. The possession of such a knowledge may afterwards prove of great value if he happens to settle in some district where the other medical men have had no experience of it. The out-patient department, if not wholly under his charge, is partly so, and in any case he has to attend to all casualties. Another duty that sometimes falls to the lot of a resident in a provincial hospital is lecturing to the probationer nurses, and this will afford him good training in drawing up a syllabus and in preparing the lectures, and will refresh his knowledge of anatomy and physiology. The visiting staff are generally selected from the best men in the district, and, as they are usually in general practice, they mirror the customs of such practice. The accusation that good surgery is not seen in the smaller hospitals is not strictly accurate.

The resident officer, if he so desires, may get a good deal of minor surgery, such as falls to the lot of the assistant surgeon in a large city hospital, to do. Should the resident be anxious to test some new drug or try some new treatment, he will as a rule find that the staff will give him liberty to do so, and will be interested to hear of his results.

In spite of his multifarious duties, the resident in such a hospital has, as a rule, sufficient leisure, and the visiting staff are generally only too glad and willing to arrange to allow him an occasional day off. Socially life in a small provincial town is very pleasant, and the resident finds that many friends are made and many happy evenings spent. The salary attached to such posts is by no means the least important point. This varies from £50 to £100 per annum, with board, lodging, and washing, and the young practitioner becomes self-supporting.

It will thus be seen that something can be said in favour of both courses; but if a man intends subsequently to engage in general practice a year or two in a provincial hospital is preferable to spending the same time in a city hospital. The more varied character of the work, and the fact that the cases seen and treated are more likely to be of the nature of those liable to be afterwards encountered in general practice, make such an appointment a valuable practical training school for the future general practitioner. It is a well-known fact that rare and interesting cases tend to gravitate to a city hospital, and while of value to those collecting such cases, they are really of little value to a general practitioner. The graduate who aims at consulting practice in the future will find the work, experience, and training of a city hospital invaluable.

